# A real-time PCR tool for the surveillance of zoonotic *Onchocerca lupi* in dogs, cats and potential vectors

**DOI:** 10.1371/journal.pntd.0006402

**Published:** 2018-04-04

**Authors:** Maria Stefania Latrofa, Giada Annoscia, Vito Colella, Maria Alfonsa Cavalera, Carla Maia, Coralie Martin, Jan Šlapeta, Domenico Otranto

**Affiliations:** 1 Department of Veterinary Medicine, Università degli Studi di Bari, Valenzano, Italy; 2 Global Health and Tropical Medicine (GHTM), Instituto de Higiene e Medicina Tropical (IHMT), Universidade Nova de Lisboa (UNL), Rua de Junqueira, Lisboa, Portugal; 3 Unité Molécules de Communication et Adaptation des Microorganismes (MCAM, UMR 7245), Sorbonne Universités, Muséum National d'Histoire Naturelle, CNRS, Paris, France; 4 Sydney School of Veterinary Science, Faculty of Science, The University of Sydney, Sydney, New South Wales, Australia; University of Georgia, UNITED STATES

## Abstract

The ocular onchocercosis is caused by the zoonotic parasite *Onchocerca lupi* (Spirurida: Onchocercidae). A major hindrance to scientific progress is the absence of a reliable diagnostic test in affected individuals. Microscopic examination of skin snip sediments and the identification of adults embedded in ocular nodules are seldom performed and labour-intensive. A quantitative real-time PCR (qPCR) assay was herein standardized for the detection of *O*. *lupi* DNA and the results compared with microscopic examination and conventional PCR (cPCR). The specificity of qPCR and cPCR was assessed by processing the most common filarial nematodes infecting dogs, skin samples from *O*. *lupi* infected (n = 35 dogs) or uninfected animals (n = 21 dogs; n = 152 cats) and specimens of potential insect vector (n = 93 blackflies; n = 59 mosquitoes/midges). The analytical sensitivity of both assays was assessed using 10-fold serial dilutions of DNA from adult specimen and from a pool of microfilariae. The qPCR on skin samples revealed an analytical specificity of 100% and a sensitivity up to 8 x 10^−1^ fg/2μl *O*. *lupi* adult-DNA and up to 3.6 x 10^−1^ pg/2μl of mfs-DNA (corresponding to 1 x 10^−2^ mfs/2μl). Only 9.5% *O*. *lupi*-infected skin samples were positive for cPCR with a sensitivity of 8 x 10^−1^ pg/2μl of DNA. Out of 152 blackflies and mosquitoes/midges, eight specimens experimentally infected (n = 1 *S*. *erythrocephalum*; n = 1 *S*. *ornatum*; n = 6 *Simulium* sp.) were positive by qPCR. The qPCR assay herein standardized represents an important step forward in the diagnosis of zoonotic onchocercosis caused by *O*. *lupi*, especially for the detection and quantification of low number of mfs. This assay provides a fundamental contribution for the establishment of surveillance strategies aiming at assessing the presence of *O*. *lupi* in carnivores and in insect species acting as potential intermediate hosts. The *O*. *lupi* qPCR assay will enable disease progress monitoring as well as the diagnosis of apparently clinical healthy dogs and cats.

## Author summary

The diagnosis of zoonotic ocular onchocercosis caused by *Onchocerca lupi* (Spirurida: Onchocercidae) is currently based on microscopic examination of skin snip sediments and on the identification of adults embedded in ocular nodules. These methods are labour-intensive and require multiple steps to achieve the diagnosis. In this context, a novel quantitative real-time PCR assay (qPCR) has been herein standardized and analytical specificity and sensitivity assessed. The results indicate that the qPCR assay could represent an important step forward in the diagnosis of onchocercosis, in carnivores and in insect species acting as potential intermediate hosts.

## Introduction

Within the genus *Onchocerca* (Spirurida: Onchocercidae), *Onchocerca volvulus* and *Onchocerca lupi* parasitize humans and carnivores, respectively [1–5], the latter being a zoonotic agent [6,7]. While *O*. *volvulus* is a well-known parasite of humans transmitted by blackflies (*Simulium* spp.) [8,9], the epidemiology of *O*. *lupi* is far from being understood, particularly because the information about insect species acting as vectors is lacking. Only *Simulium tribulatum* was suggested as the putative vector of this filarial worm in California (USA), but proof of its intermediate host competence is currently absent [10]. *Onchocerca lupi* belongs to the spirurids in the Nematode clade III [11] was first detected from a Caucasian wolf (*Canis lupus*) in Georgia [12], and, only recently, diagnosed in dogs and cats from Europe (Greece, Portugal, Spain, Germany, Hungary) and USA [13–20]. The reports of *O*. *lupi* infection are mainly based on the presence of ocular nodules on the eyelids, conjunctiva, and sclera [3,21,22], though the localization of adult worms in the retrobulbar area of the canine patients may impair the assessment of its distribution in endemic areas [23]. The detection of microfilariae (mfs) in skin snip sediments is the only available tool for the diagnosis of the infection when nodules are not apparent in the eyes. The retrieval and identification of mfs in skin snip samples is a rather invasive and time-consuming method, highly dependent on the anatomical location of skin biopsy and mfs density [24]. Again, the detection of mfs may depend upon the prepatent period, previous microfilaricidal treatments, and on the operator’s skills in examining skin sediments, as described for *O*. *volvulus* [25,26].

Conventional PCR (cPCR) amplification and sequencing of mitochondrial NADH dehydrogenase subunit 5 (ND5) and cytochrome *c* oxidase subunit 1 (*cox*1) genes are available for the molecular identification of *O*. *lupi* adults and mfs [7,27,28]. The cPCR, however, may be relatively labour-intensive and exhibit low sensitivity, mainly for mfs detection, limiting the establishment of large-scale epidemiological studies in vertebrate hosts and putative vectors.

Here, we developed a quantitative real-time PCR (qPCR) assay based on the hybridization probe to detect *O*. *lupi* DNA in host and putative vector samples. The diagnostic validity of qPCR assay was compared with microscopic examination and cPCR methods.

## Methods

### Ethics statement

All dogs’ and cats’ skin samples were collected in previous studies [17,29] and approved by the ethical committee of the Department of Veterinary Medicine of the University of Bari (Prot. Uniba 1/16) and by the ethical committee of the Faculty of Veterinary Medicine, Universidade Lusófona de Humanidades e Tecnologias.

### Samples

Genomic DNA of adult specimens of *O*. *lupi* (n = 3), as well as DNA from single (n = 7) or pooled mfs (n = 10), collected from dogs in different geographical locations (Table 1) were used as control. All specimens were previously identified based on morphological and molecular analyses [18,30].

**Table 1:** Filarial nematodes used to assess the analytical specificity of the qPCR assay.

Species	Host	Collection locality	Source	ID sample
*Onchocerca lupi*	*Canis lupus familiaris*	USA (Minnesota)	Adult	100–14
	*Canis lupus familiaris*	USA (New Mexico)	Adult	132–14
	*Canis lupus familiaris*	USA (Colorado)	Adult	478–15
	*Canis lupus familiaris*	Portugal	Microfilariae	63–12
	*Canis lupus familiaris*	Greece	Microfilariae	62–12
	*Canis lupus familiaris*	Portugal	Skin	537–15
	*Felis catus*	Portugal	Skin	61–15
*Onchocerca armillata*	*Bos taurus*	Cameroon	DNA	54FKA2
*Onchocerca bohemi*	*Equus caballus*	Italy	Adult	409
*Onchocerca fasciata*	*Camelus dromedaries*	Iraq	Adult	200
*Onchocerca gutturosa*	*Bos taurus*	Cameroon	DNA	54FKG1
*Onchocerca ochengi*	*Bos taurus*	Cameroon	DNA	54FKO2
*Dirofilaria immitis*	*Vulpes vulpes*	Italy	Adult	377
*Dirofilaria repens*	*Canis lupus familiaris*	Italy	Adult	379
*Cercopithifilaria grasii*	*Canis lupus familiaris*	Portugal	Skin	81–16
*Cercopithifilaria bainae*	*Canis lupus familiaris*	Portugal	Skin	81–16
*Cercopithifilaria* sp. II	*Canis lupus familiaris*	Portugal	Skin	81–16
*Acanthocheilonema reconditum*	*Canis lupus familiaris*	Italy	Blood	496
*Brugia malayi*	*Meriones unguiculatus* (experimental cycle)	FR3 strain	DNA	8YT1
*Brugia pahangi*	*Meriones unguiculatus* (experimental cycle)	FR3 strain	DNA	46YT
*Wuchereria bancrofti*	*Homo sapiens*	Singapore	DNA	82YT FIL13/01

### Primers and probe design and qPCR run protocol

Primers (*O*.*l*.F 5′-GGAGGTGGTCCTGGTAGTAG-3′; *O*.*l*.R 5′- GCAAACCCAAAACTATAGTATCC-3′) and a TaqMan-MGB hydrolysis probe (FAM-5’-CTTAGAGTAGAGGGTCAGCC-3’-non-fluorescent quencher-MGB; Applied Biosystems; Foster City, CA, USA), targeting partial *cox*1 gene (90bp), were designed by alignment of sequences from a wide range of closely related filarial nematodes available from GenBank database (Table 2), using Primer Express 2.0 (Applied Biosystems, Foster City, CA). Specificity of the primers and probe for *O*. *lupi* were confirmed *in silico* using the basic local alignment search tool (BLAST, GenBank, NCBI).

**Table 2:** GenBank accession numbers (AN) of mitochondrial cytochrome *c* oxidase subunit 1 sequences of filarial nematodes used for primers and TaqMan-probe design.

Parasite	AN	Host	Collection locality
*Onchocerca lupi*	KC686702	*Canis lupus familiaris*	Greece
	KC686701	*Canis lupus familiaris*	Portugal
	EF521408	*Canis lupus familiaris*	Hungary
*Onchocerca armillata*	KP760200	*Bos taurus*	Cameroon
*Onchocerca boehmi*	KX898458	*Equus caballus*	Italy
*Onchocerca dewittei japonica*	AM749267	*Sus scrofa leucomystax*	Japan: Oita
*Onchocerca eberhardi*	AM749268	*Cervus nippon*	Japan: Oita
*Onchocerca ochengi*	KC167350	*Simulium damnosum* sensu lato	Cameroon: northern
*Onchocerca gibsoni*	AJ271616	*Bos taurus*	Australia: Queensland
*Onchocerca gutturosa*	KP760201	*Bos taurus*	Cameroon
*Onchocerca lienalis*	KX853326	*Bos taurus*	United Kingdom: Wales
*Onchocerca ramachandrini*	KC167356	*Simulium damnosum* sensu lato	Cameroon: northern
*Onchocerca suzukii*	AM749277	*Nemorhaedus crispus*	Japan: Yamagata
*Onchocerca skrjabini*	AM749269	*Cervus nippon*	Japan: Oita
*Onchocerca* sp. ‘Siisa’	KC167354	*Simulium damnosum* sensu lato	Cameroon: northern
*Onchocerca volvulus*	KC167355	*Simulium damnosum* sensu lato	Cameroon: northern
*Brugia malayi*	KP760171	*Meriones unguiculatus*	FR3 strain
*Brugia pahangi*	EF406112	*Homo sapiens*	Malaysia
*Wuchereria bancrofti*	AM749235	*Homo sapiens*	Italy
*Cercopithifilaria bainae*	JF461457	*Canis lupus familiaris*	Italy: Sicily
*Cercopithifilaria bulboidea*	AB178834	*Cervus nippon*	Japan
*Cercopithifilaria crassa*	AB178840	*Cervus nippon*	Japan
*Cercopithifilaria grassii*	JQ837810	*Canis lupus familiaris*	Italy
*Cercopithifilaria japonica*	AM749263	*Ursus thibetanus*	Japan: Gifu
*Cercopithifilaria longa*	AB178843	*Cervus nippon*	Japan
*Cercopithifilaria minuta*	AB178846	*Cervus nippon*	Japan
*Cercopithifilaria multicauda*	AB178848	*Cervus nippon*	Japan
*Cercopithifilaria rugosicauda*	KF479370	*Capreolus capreolus*	Italy
*Cercopithifilaria* sp. II	JQ837809	*Canis lupus familiaris*	Italy
*Cercopithifilaria shohoi*	AB178850	*Cervus nippon*	Japan
*Cercopithifilaria tumidicervicata*	AB178852	*Cervus nippon*	Japan
*Acanthocheilonema delicata*	JQ289993	*Meles anakuma*	Japan
*Acanthocheilonema odendhali*	KF038145	*Callorhinus ursinus*	USA: Alaska
*Acanthocheilonema reconditum*	JF461456	*Canis lupus familiaris*	Italy: Sicily
*Acanthocheilonema spirocauda*	KF038155	*Erignathus barbatus*	USA: Alaska
*Acanthocheilonema vitaea*	KP760169	*Meriones unguiculatus*	FR3 strain
*Dirofilaria immitis*	EU169124	*Ailurus fulgens*	China
*Dirofilaria repens*	AM749230	*Canis lupus familiaris*	Italy

qPCR reactions were carried out in a final volume of 20μl, consisting of 10μl of IQ Supermix (Bio-Rad Laboratories, Hercules CA, USA), 7.1μl of Di-Ethyl Pyro-Carbonate (DEPC) treated pyrogen-free DNase/RNase-free water (Invitrogen, Carlsbad, CA, USA), 2μl of template DNA (except no-template controls), 5 pmol and 0.5 pmol for primers and probe, respectively.

The run protocol consisted of a hot-start at 95°C for 3 min, and 40 cycles of denaturation (95°C for 10 sec) and annealing-extension (64°C for 30 sec). All assays were carried out in duplicate and a no-template control was included in each run. The qPCR was performed in a CFX96 Real-Time System (Bio-Rad Laboratories, Inc., Hercules CA, USA) and the increase in the fluorescent signal was registered during the extension step of the reaction and analysed by the CFX Manager Software Version 3.1 (Bio-Rad).

### Specificity and sensitivity of qPCR

To investigate the analytical specificity of the assay, genomic samples of *Onchocerca* spp. and of the most common filarial nematodes infesting dogs (Table 1) were used. The specificity of the assay was tested by using DNA from skin samples of naturally infected dogs, which were positive for *O*. *lupi* (n = 35) at microscopic examination [29]. Skin samples were divided in five groups (G1-G5) according to their mfs load (Table 3), being 14 also co-infected with *Cercopithifilaria bainae* and *Cercopithifilaria* sp. II. Skin samples (dogs n = 21; cats n = 152), which did not test positive to any mfs [17,29], were used as negative control.

**Table 3:** Skin samples tested for *Onchocerca lupi* by qPCR, divided (Groups 1–5) according to the parasitic load (mfs) microscopically detected. The mean, minimum, maximum and standard deviation (sd) values of the threshold cycle (Cq), parasite load (Starting Quantity (SQ) value, expressed as ng/μl of DNA for reaction) and microfilariae concentration, assessed by qPCR is reported.

Parasitic load (mfs)		Skin (n)	Cq			Mfs			DNA		
						SQ			SQ		
			Mean	Min-Max	SD	Mean	Min/Max	SD	Mean	Min/Max	SD
G1	1 < 5	16	33.49	32.12–35.89	1.2	1.9	2.6 x 10−1–3.8	1.2	6.1 x 10^−2^	9.5 x 10−3–1.3 x 10^−1^	4.3 x 10^−2^
G2	6 < 10	7	31.24	30.24–31.75	0.5	6.9	4.9–10	1.8	2.5 x 10^−1^	1.8 x 10−1–3.8 x 10^−1^	6.5 x 10^−2^
G3	11 < 25	8	29.92	29.06–31.3	0.8	19.1	6.7–29.8	8.6	6.9 x 10^−1^	3.1 x 10−1–1.1	3 x 10^−1^
G4	26 < 40	2	28.65	28.37–28.93	0.4	38.4	3.5 x 10^1^–4.1 x 10^1^	4	1.4	1.3–1.5	1.5 x 10^−1^
G5	> 40	2	27.52	27.41–27.63	0.1	96	1 x 102–8.9 x 10^1^	10.2	3.4	3.2–3.7	3.7 x 10^−1^

Specimens of blackflies (n = 66) and mosquitoes/midges (n = 39) collected from 2011 to 2014 in Greece [31], and 27 blackflies and 20 *Aedes albopictus* (colony specimens) experimentally infected by intrathoracic microinjection with mfs of *O*. *lupi* (parasitic load of 20mfs/μl) were analyzed after death (i.e., from one to 10 days post infection) (Table 4).

**Table 4:** Blackflies and mosquitoes/midges specimens used to test the analytical specificity of qPCR assay.

Geographical origin	Blackflies species	Number (pos/tot)	Mosquitoes/ midges species	Number (pos/tot)
Greece	*Simulium balcanicum*	0/14	*Culex pipiens pipiens*	0/10
	*Simulium erythrocephalum*	0/6	*Ochlerotatus caspius*	0/10
	*Simulium pseudequinum*	0/10	*Anopheles maculipennis*	0/4
	*Simulium reptans*	0/23	*Coquilletidia richiardii*	0/2
	*Simulium ornatum*	0/4	*Culiseta annulata*	0/3
	*Simulium velutium*	0/9	*Culicoides* spp.	0/1
			Ceratopogonidae	0/5
			Psychodidae	0/4
Italy				
Basilicata region	*Simulium erythrocephalum*	1/1*		
	*Simulium linatum*	0/5*		
	*Simulium ornatum*	1/4*		
	*Simulium pseudequinum*	0/10*		
	*Simulium* sp.	6/7*		
Reggio Emilia			*Aedes albopictus*	0/20*
Total		8/93		0/59

* = Specimens experimentally infected by intrathoracic microinjection with microfilariae of *Onchocerca lupi*.

The analytical sensitivity of the qPCR assay was assessed using 10-fold serial dilutions of DNA from adult specimen (i.e., ranging from 8 × 10^4^ to 8 × 10^−3^ fg/2μl of reaction) and from a pool of 10 mfs (i.e., ranging from 10 to 10 × 10^−3^ microfilariae/2μl of reaction, corresponding to 3.6 ×10^−1^ ng/2μl to 3.6 ×10^1^ fg/2μl of DNA). Ten replicates of each serial dilution were submitted to the same run for assessment of intra-assay reproducibility.

Genomic DNA was isolated from all skin samples and from *O*. *lupi* adults and mfs, blackflies, mosquitoes and midges specimens using the commercial kits DNeasy Blood & Tissue Kit (Qiagen, GmbH, Hilden, Germany), respectively, following the manufacturers’ instructions. The amounts of purified DNA were determined spectrophotometrically using the Qubit (Applied Biosystems, Foster City, CA, USA).

### Specificity and sensitivity of cPCR

The analytical specificity and sensitivity of the cPCR for the specific amplification of *cox*1 gene fragment (∼689bp; [32]) was assessed by testing genomic DNA of: i) skin samples with different parasitic load of *O*. *lupi* (Table 3), ii) serial dilution of *O*. *lupi* mfs DNA (i.e., from 3.6 ×10^1^ pg/2μl to 3.6 ×10^−3^ pg/2μl) and iii) DNA of adult specimens (i.e., from 8 ×10^1^ ng/2μl to 8 x 10^−3^ fg/2μl).

All cPCR products were resolved in 0.5x GelRed stained (Biotium, CA, USA) agarose gels (2%), purified using enzymatic purification (Exo I-FastAP; Thermo Fisher Scientific, MA, USA) and sequenced in an automated sequencer (3130 Genetic Analyzer). All sequences generated were compared with those available in GenBank using Basic Local Alignment Search Tool (BLAST) [33].

## Results

All *O*. *lupi* naturally-infected dog positive at skin samples examination by microscopy, considered the gold standard method as true positives, were positive by the *O*. *lupi* qPCR herein assessed (specificity of 100%). Out of 21 skin samples microscopically and qPCR positive for *O*. *lupi*, two were positive by cPCR (parasite load of 8 and 25 mfs), revealing a low analytical cPCR specificity (i.e., 9.5%). None of cat’s skin samples were positive by qPCR.

**Fig 1:**
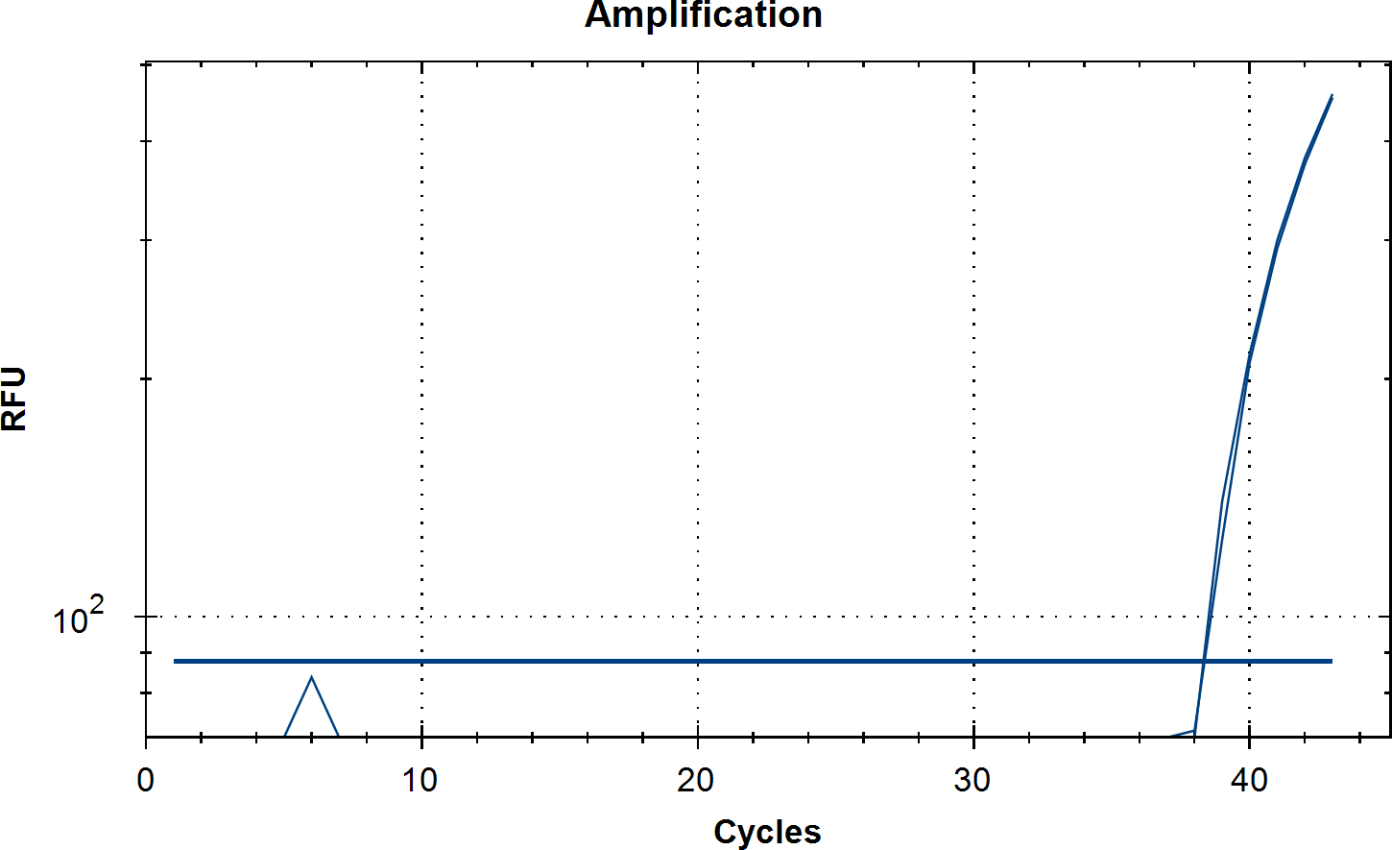
Assessment of the specificity of qPCR assay in the detection of *Onchocerca lupi* DNA. The amplification plot is represented by the fluorescent signal accordingly to relative fluorescence units (RFU) and threshold cycle.

A specific fluorescent signal was recorded for all *O*. *lupi* adult and mfs positive controls tested (Fig 1). No fluorescence was obtained for all other *Onchocerca* species or filarial nematodes examined as well as for skin samples used as negative control.

**Fig 2:**
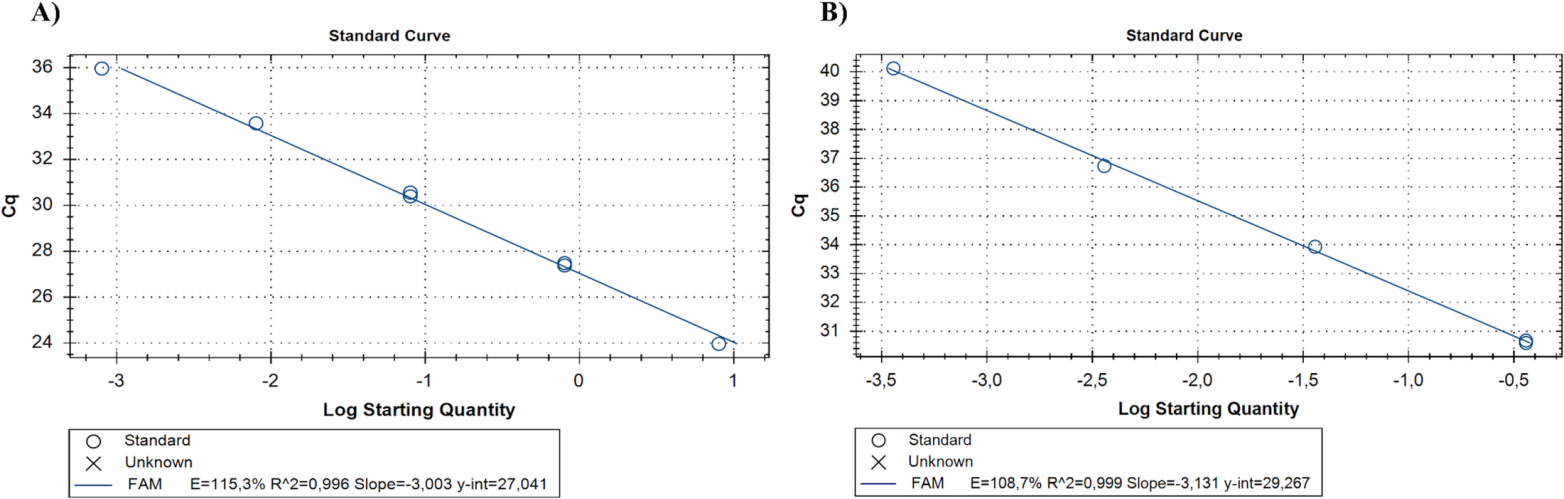
Standard curves generated from serial dilutions of (A) genomic DNA from adult (from 8 × 10^4^ to 8 × 10^−3^ fg/2μl of reaction) and microfilariae (B) (from 3.6 ×10^−1^ ng/2μl to 3.6 ×10^1^ fg/2μl of reaction) of *Onchocerca lupi*. Each point was tested in triplicate. Slope, efficacy and *R*^2^ are reported on the bottom.

**Fig 3:**
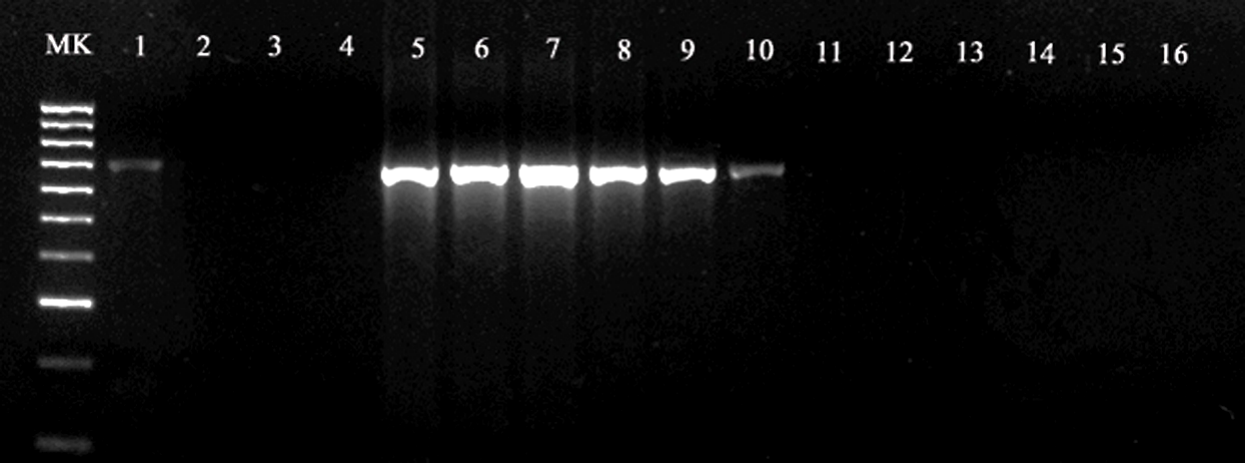
Detection limit of the conventional PCR assay determined by 10-fold serial dilution of genomic DNA of microfilariae and adult of *Onchocerca lupi*. Lanes 1–4, from 3.6 ×10^1^ pg/2μl to 3.6 ×10^−3^ pg/2μl of *O*. *lupi* mfs DNA (i.e., from 1 to 1×10^−4^ mfs); Lanes 5–15, from 8 ×10^1^ ng/2μl to 8 x 10^−3^ fg/2μl of *O*. *lupi* adult DNA; Line 16, no-DNA control; M, 100 bp DNA marker.

The analytical sensitivity of qPCR was confirmed by detection of up to 8 x 10^−1^ fg/2μl and 3.6 x 10^−1^ pg/2μl of DNA (i.e., corresponding to 1 x 10^−2^ mfs/2μl) of *O*. *lupi* adult worm and mfs, respectively (Fig 2A and 2B). qPCR efficiencies ranged from 108.7 to 115.3% with an *R*^2^ from 0.996 to 0.999 and Slope from -3.003 to -3.131, for both adult and mfs (Fig 2A and 2B). The mean parasite load detected for the positive skin samples, ranged from 1.9 to 96 mfs/2μl of reaction, corresponding to 6.1 x 10^−2^ ng/2μl (mean cycle threshold of 33.49) and to 3.4 ng/2μl DNA (mean cycle threshold of 27.52), respectively (Table 3). The results of mfs detection by qPCR overlapped the values obtained by the microscopic examination. The detection limit registered for cPCR was up to 8 x 10^−1^ pg/2μl for adult worms and up to 3.6 x 10^1^ pg/2μl for mfs DNA (i.e., corresponding to 1 mf/2μl), respectively (Fig 3).

Out of 152 blackflies, mosquitoes and midges, eight *Simulim* spp. (n = 1 *S*. *erythrocephalum*; n = 1 *S*. *ornatum*; n = 6 *Simulium* sp.), experimentally infected and died from 1 to three days post infection, returned positive signal for *O*. *lupi* DNA (Table 4). All field-collected blackflies and mosquitoes were negative for *O*. *lupi* DNA using qPCR (Table 4). All blackflies positive for qPCR scored positive also for cPCR.

Sequences derived from all amplicons of cPCR matched with 99–100% nucleotide identity appropriate reference sequences of *O*. *lupi* available from GenBank (accession numbers KC686702, KC686701).

## Discussion

A qPCR assay has been developed for the detection of *O*. *lupi* in animal skin snip samples and potential vectors and proved to be a sensitive and specific tool for the diagnosis of this parasite, with a mean detection limit as low as 1.9 mfs per reaction. In addition, the high sensitivity of the qPCR protocol has been demonstrated by detecting a small amount of DNA (up to 8 x 10^−1^ fg/2μl for adult and up to 3.6 x 10^−1^ pg/2μl for mfs), by the slope value of standard curve (−3.131), the efficiency (115.3%) and the coefficient of determination (*R*^2^ = 0.999). These features of the assay are due to the selection of a stable hydrolysis probe designed (100% specific for *O*. *lupi* DNA), as well as to the choice of the target gene used. Indeed, *cox*1 gene of the mitochondrial DNA has been well recognized as a “barcode” for filarial nematodes [34], with a high amplification efficiency, also due to the large copy numbers enabling the detection of minimum amounts of DNA [35–37]. Though few *Onchocerca* species DNA were herein tested, which may represent a limitation of the qPCR assay, this new tool provides an alternative to the labor intensive microscopic examination of skin snip samples and to cPCR for the diagnosis of *O*. *lupi* [38]. The qPCR assay was highly specific in revealing *O*. *lupi* DNA both in co-infected samples from dogs as well as in potential vector species, avoiding the sequencing confirmation needed using cPCR with filarioid generic primers [32]. Overall, the positive fluorescent signal from samples of *O*. *lupi*, from different geographical areas (i.e., Europe and USA), which displayed genetic intraspecific variability [18], indicates the usefulness of the qPCR also for the surveillance of *O*. *lupi* where the parasite has been reported [13,14,16,17,19,39–41]. Similarly, even if the qPCR cannot discriminate between viable and nonviable parasites or immature and infective larvae, the assay could be useful for detecting *O*. *lupi* in blackfly, mosquito and/or midge species, potentially involved in the transmission of this parasite. Indeed, the specificity of the qPCR to amplify exclusively the DNA of the pathogen in potential insect vectors herein tested, may ultimately assist in the quest to identify the elusive vector of *O*. *lupi*.

The newly designed assay represents an improvement in the diagnosis of onchocercosis, by the detection and quantification of low mf densities from tissue samples and could provide a contribution to disease progress monitoring and to the surveillance of *O*. *lupi*-infected dogs, avoiding the introduction and/or spread of this life-threatening parasitic nematode, as well as to the identification of apparently healthy animals [29, 42].

The qPCR may speed-up time of diagnosis and prompt treatments of infected animals, which may avoid the appearance of nodular lesions in the eyes or in other anatomical localizations [43].

A TaqMan-based specific and sensitive assay without sequencing is expected to assist high-throughput analysis of samples, eventually leading to improve disease monitoring under the frame of a Public Health perspective. This would be particularly relevant considering that, since its first description of its zoonotic potential [7], cases of zoonotic onchocercosis are being detected increasingly in people from Europe, Iran and the USA [44–47].
